# Anti-Tumor Mechanisms Associated With Regulation of Non-Coding RNA by Active Ingredients of Chinese Medicine: A Review

**DOI:** 10.3389/fonc.2020.634936

**Published:** 2021-02-18

**Authors:** Tian-Jia Liu, Shuang Hu, Zhi-Dong Qiu, Da Liu

**Affiliations:** School of Pharmacy, Changchun University of Chinese Medicine, Changchun, China

**Keywords:** microRNA, lncRNA, non-coding RNA, active ingredients of Chinese medicine, anti-cancer

## Abstract

Cancer has become the second leading cause of death worldwide; however, its complex pathogenesis remains largely unclear. Previous research has shown that cancer development and progression are closely associated with various non-coding RNAs, including long non-coding RNAs and microRNAs, which regulate gene expression. Target gene abnormalities are regulated and engaged in the complex mechanism underlying tumor formation, thereby controlling apoptosis, invasion, and migration of tumor cells and providing potentially effective targets for the treatment of malignant tumors. Chemotherapy is a commonly used therapeutic strategy for cancer; however, its effectiveness is limited by general toxicity and tumor cell drug resistance. Therefore, increasing attention has been paid to developing new cancer treatment modalities using traditional Chinese medicines, which exert regulatory effects on multiple components, targets, and pathways. Several active ingredients in Chinese medicine, including ginsenoside, baicalin, and matrine have been found to regulate ncRNA expression levels, thus, exerting anti-tumor effects. This review summarizes the scientific progress made regarding the anti-tumor mechanisms elicited by various active ingredients of Chinese medicine in regulating non-coding RNAs, to provide a theoretical foundation for treating tumors using traditional Chinese medicine.

## Introduction

The incidence of cancer has tripled over the past three decades and is expected to increase five-fold by 2030 (1). Nevertheless, the precise mechanism associated with cancer pathogenesis remains largely unclear as it is highly complex and diverse. Since the 1970s, gene therapy has become an increasingly attractive strategy for the treatment of various diseases. Accordingly, more recently, the focus has shifted toward identifying specific genetic etiologies of cancer to design effective gene therapies to overcome the challenges associated with traditional cancer treatment modalities. As the human genome is gradually deciphered, RNA has been shown to play an auxiliary role as an intermediate vector of genetic information, and an increasing number of regulatory functions have been ascribed to this class of molecules. In particular, non-coding sequences, which account for 99% of the total human genome, have received greater attention (2). Most of the identified non-coding RNAs (ncRNAs), including long non-coding (lnc)RNAs, micro (mi)RNAs, small interfering (si)RNAs, Piwi-interacting (pi)RNAs, ribosomal (r)RNAs, transfer (t)RNAs, and small nuclear (sn)RNAs, participate in the translation, modification, and other cellular functions. Among these molecules, lncRNAs and miRNAs have been researched more intensively to decipher their fundamental roles in many diseases, including cancer (3). In fact, lncRNAs were initially believed to be the “noise” of genome transcription, a by-product of RNA polymerase II transcription, and were considered biologically non-functional (4). However, recent studies have shown that lncRNAs play an essential role in cancer signal transduction pathways by interacting with proteins and RNA (5) and are reportedly associated with various cancers, including those of the stomach (6–8), lung (9, 10), breast (11, 12), and prostate (13, 14). Specifically, inhibition of lncRNA H19 and lncRNA *PVT1* expression can effectively inhibit the cancerization, metastasis (15), and angiogenesis (7) of gastric cancer.

miRNA is a class of small (18–22 nucleotides) ncRNA molecules; it is present in all eukaryotic cells, with over 2,000 of these RNAs being identified in humans (3). However, ncRNAs have also been described in insects, plants, fungi, bacteria, and viruses (16, 17). Compared with lncRNA, the regulatory mechanism of miRNA is relatively simple and clear. It regulates gene expression by either suppressing mRNA translation or degrading mRNA molecules (18). Previous studies have found that the abnormal expression of miRNAs can disrupt many signaling pathways resulting in reduced proliferation, migration, and invasion of cancer cells and the promotion of apoptosis (19–21). Numerous carcinogenic miRNAs, including miR-638, miR-155, miR-31, miR-21, miR-221, miR-222, and miR-294 among others, are overexpressed in various malignant tumors (22–24), whereas others, including miR-429, miR-211, miR-1271, and miR-34a, exert anti-cancer effects and are under-expressed in cancer cells (25, 26).

The emergence of ncRNA provides new alternatives for cancer treatment, but its effective application in clinical cancer treatment remains challenging. At present, chemotherapy is one of the most important means of treating malignant tumors. However, its efficacy is limited by systemic toxicity and tumor cell resistance (27). Traditional Chinese medicine has been practiced for thousands of years because of its safety and minimal side effects. To date, many cancer patients have used traditional Chinese medicine as an alternative therapy for cancer treatment (28). Moreover, some of the active ingredients of Chinese medicine have been shown to exert anti-cancer effects by regulating ncRNAs and acting on various signaling pathways and cancer-related molecular targets, thus, inhibiting tumor proliferation, metastasis, and invasion and inducing cancer cell apoptosis (29). For instance, the isoflavone calycosin inhibits nasopharyngeal carcinoma cell growth by regulating the lncRNA *EWSAT1* and its downstream *TRAF6* pathways (30). Additionally, curcumin upregulates miR-145 expression to inhibit cell proliferation and invasion *in vitro*, while inducing cell cycle arrest (31). These reports suggested that ncRNA have anti-tumor abilities that regulate cancer cell apoptosis, proliferation, resistance, metastasis, and invasion.

## Active Ingredients of Traditional Chinese Medicine Regulate the Anti-Tumor Mechanism of miRNA

Over the past decades, miRNA has been shown to play a regulatory role in many cancer signaling pathways (32). The active ingredients in traditional Chinese medicine act through different mechanisms to upregulate the expression of tumor-suppressing miRNAs and downregulate the expression of oncogenic miRNAs (33). They also inhibit tumor occurrence and development by inducing cell apoptosis to inhibit tumor metastasis, enhancing cell cycle arrest to reduce drug resistance, and by downregulating other pathways (34) (**Table 1**).

**Table 1:** Detailed information on Chinese medicine active ingredients targeting miRNAs.

Active Compound	Types of cancer	miRNA	Target Genes	Related Hallmark	Reference
Ginsenoside Rh2	Acute myeloid leukemia	miR-21	Bcl-2	Induce apoptosis	(35)
Ginsenoside Rh2	Human liver cancer	miR-21	MCL1/Nrf2/Bcl-2	Induce apoptosis	(36)
Ginsenoside Rh2	Prostate cancer	miR-4295	CDKN1A	Anti-proliferation	(37)
Ginsenoside Rh2	Lung adenocarcinoma	miR-491	MMP-9	Anti-metastasis	(38)
Matrine	Human thyroid cancer	miR-21	PTEN/p-Akt	Induce apoptosis	(39, 40)
Honokiol	Osteosarcoma	miR-21	PTEN/PI3K/AKT	Induce apoptosis	(41)
Berberine	Non-small cell lung cancer	miR-19a	TF/MAPK	Induce apoptosis	(42)
Baicalin	Colon cancer	miR-217	DKK1	Induce apoptosis	(33)
Baicalin	Hepatoma	miR-3127-5p	PI3K/Akt	Cell cycle arrest Anti-proliferation	(43)
Resveratrol	Breast cancer	miR-122-5p/miR-542-3p	XIAP/Bcl-2	Induce apoptosis	(44)
Resveratrol	Acute lymphoblastic leukemia	miR-196b/miR-1290	Caspase-3	Induce apoptosis/anti-migration/cycle arrest	(45)
Resveratrol	Cancer	miR-326	PKM2	Induce apoptosis	(46)
Resveratrol	Breast cancer	miR-34a/miR-424/miR-503	Bcl2/p53	Anti-proliferation	(47)
Triacetyl resveratrol	Pancreatic cancer	miR-200	Shh	Anti-MET process/Anti-metastasis	(48)
Resveratrol	Low invasive breast cancer	miR-122-5p	Bcl2/CDKs	Enhance chemosensitivity	(49)
Resveratrol	Colorectal cancer	miR-200c	Vimentin/ZEB1	Anti-MET process/anti-metastasis	(50)
Ginsenoside Rg3	Oral squamous cell carcinoma	miR-221	PI3K/AKT, MAPK/ERK	Anti-EMT process	(51)
Ginsenoside Rg3	Ovarian cancer	miR-145	DNMT3A/FSCN1	Anti-EMT process	(52)
Camptothecin	Cancer	miR-125b	Bak1/Mcl1/p53	Induce apoptosis	(53)
Camptothecin	Cervical cancer	miR-15a/16	Rictor	Enhance chemosensitivity	(54)
Paeoniflorin	Gastric carcinoma	miR-124	PI3K/Akt/STAT3	Anti-proliferation	(55)
Paeoniflorin	Human glioma cells	miR-16	MMP-9	Anti-proliferation/induce apoptosis	(56)
Paeoniflorin	Multiple myeloma	miR-29b	MMP-2	Anti-proliferation/induce apoptosis	(57)
Shikonin	Glioblastoma	miR-143	BAG3	Induce apoptosis	(58)
Shikonin	Endometrioid endometrial cancer	miR-106b	AKT/mTOR	Anti-proliferation/Induce apoptosis	(59)
Shikonin	Gastric carcinoma	miR-195	PI3K/AKT	Anti-migration/anti-invasion	(60)
Shikonin	Cervical Cancer	miR-183-5p	E-cadherin	Anti-migration/anti-invasion	(61)
Shikonin	Retinoblastoma	miR-34a/miR-202	MYCN	Anti-proliferation	(62)
Celastrol	Ovarian carcinoma	miR-21	PI3K/p-Akt/NF-κB	Induce apoptosis	(63)
Celastrol	Colon cancer	miR-21	PI3K/AKT/GSK-3β	Anti-proliferation	(64)
Celastrol	Gastric cancer	miR-21	MMP9/Vimentin Cyclin D1/CDK6	Anti-migration/induces cell cycle arrest	(65)
Celastrol	Gastric cancer	miR-21	P27/mTOR	Induces cell cycle arrest	(66)
Celastrol	Prostate cancer	miR-101	NA	Induces autophagy	(67)
Celastrol	Prostate cancer	miR-17-92a	NA	Induces autophagy	(68)
Celastrol	Lung adenocarcinoma	miR-24/miR-181b	STAT3	Anti-proliferation	(69)
Celastrol	Lung adenocarcinoma	miR-33a-5p	mTOR/p-p70S6K/p-4EBP1	Enhance chemosensitivity	(70)

## Active Ingredients of Chinese Medicine Targeting miRNA Induce Tumor Apoptosis

**Figure 1:**
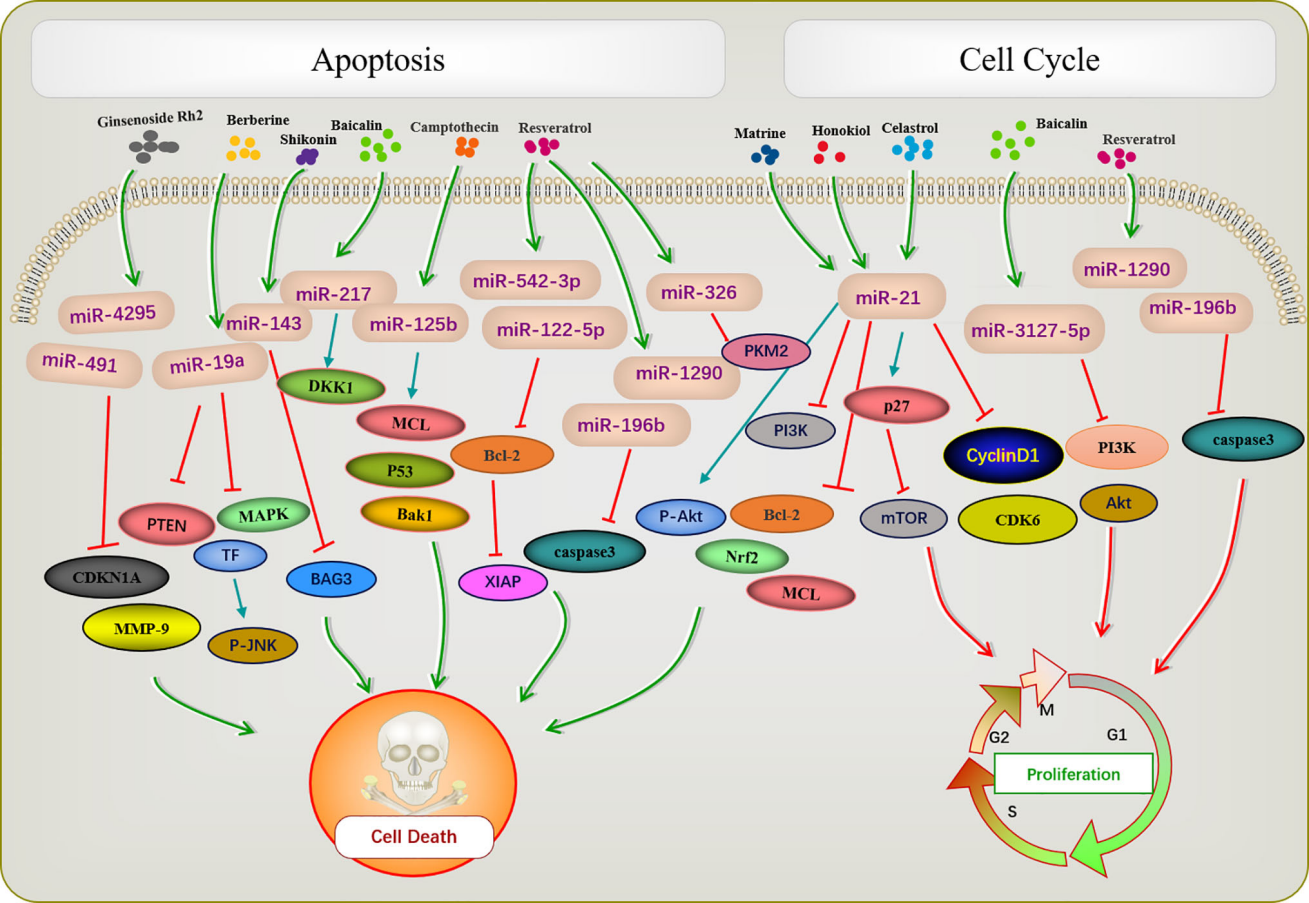
Active ingredients of Chinese medicine targeting miRNAs induce tumor apoptosis and cell cycle blocking.

Apoptosis is a basic biological phenomenon, that involves the activation, expression, and regulation of a series of genes. The active ingredients of traditional Chinese medicine regulate target genes by influencing an abnormal expression of miRNAs and inducing tumor cell apoptosis (**Figure 1**). miR-21 inhibits apoptosis in various tumor cells (71–73). Ginsenoside Rh2 is a natural monosome of ginseng total saponin, and its anti-cancer effects have been demonstrated in various tumors. A former report showed that ginsenoside Rh2 inhibited Bcl-2 by increasing miR-21 levels, which induced apoptosis and significantly decreased leukemia cell viability (35). PTEN is a classical anti-oncogene, the inhibition of PTEN is key for cell apoptosis, mainly relying on the phosphorylation and dephosphorylation of Akt. In FTC-133 human follicular thyroid cells, the alkaloid Chinese medicine active ingredient matrine is induced by upregulating the *PTEN/Akt* signaling pathway *via* the downregulating miR-21 (39) it induces apoptosis of TPC-1 human thyroid cancer cells (40). Similarly, the reverse quantitative polymerase chain reaction of an extract of *Magnolia officinalis* revealed that magnolol could induce abnormal expression of miRNA in human osteosarcoma cells, with miR-21 showing a very strong ability to downregulate mRNAs. Recent evidence has suggested that Honokiol is able to suppress the *PI3K/AKT* signaling pathway; however, it was reactivated by miR-21 overexpression. Honokiol inhibits proliferation and induces apoptosis by regulating the *miR-21/PTEN/PI3K/AKT* signaling pathway in human osteosarcoma cells (41).

A previous report described that Rh2 reduces the expression levels of MCL1 and Nrf2, suppresses colony formation, and induces HepG2 cell apoptosis by inhibiting miR-146a-5p in HepG2 cells (36). Berberine is a quaternary ammonium alkaloid isolated from *Coptis chinensis*. It inhibits the growth of non-small-cell lung cancer *via* the *miR-19a/TF/MAPK* axis and promotes apoptosis. A previous study of the mechanism of anti-tumor baicalin in human colon cancer showed that baicalin induced colon cancer cell apoptosis *via* the Wnt signaling pathway mediated by *miR-217/DDK1*, in which *DDK1* was identified as a direct downstream target gene of miR-217 (33). A previous study showed that resveratrol regulated the apoptosis and cell cycle of breast cancer cells by regulating miRNAs such as miR-125b-5p, miR-200C-3p, miR-409-3p, miR-122-5p, and miR-542-3p. Resveratrol-mediated miRNA modulation regulates key anti-apoptotic and cell cycle proteins, including *Bcl-2*, X-linked inhibitor of apoptosis protein, and CDKs, which are critical for its activity. Among these, miR-542-3p and miR-122-5P play key roles in resveratrol-mediated apoptosis of MCF-7 and MDA-MB-231 breast cancer cells, respectively (44). Resveratrol significantly reduced miR-196b/miR-1290 expression in the T-ALL (T-cell acute lymphoblastic leukemia) TTL-104 and B-ALL (B-cell acute lymphoblastic leukemia) SUP-B15 cell lines and upregulated the expression of IGFBP3. As a miR-196b/miR-1290 inhibitor, resveratrol was further demonstrated to exert antitumor effects on ALL cells including antiproliferation, cell cycle arrest, apoptosis and inhibition of migration (45). Pyruvate kinase *PKM2* is highly expressed in various tumors. The expression of miR-326 was increased after resveratrol treatment, and *miR-326/PKM2*-mediated stress and mitochondrial dysfunction were involved in apoptosis induced by resveratrol (46). Camptothecin, a cytotoxic quinoline alkaloid, is an anti-cancer compound found in plants. Deep sequencing analysis of miRNA expression profiles during the camptothecin-induced apoptosis showed that 79 miRNAs were downregulated post-treatment (74). A study of camptothecin verified that miR-125b was down-regulated in camptothecin induced apoptosis in cancer cells. Camptothecin induced apoptosis in cancer cells through miR-125b-mediated mitochondrial pathways by targeting the 3’-untranslated (UTR) regions of Bak1, Mcl1, and p53 (53). In glioblastoma stem cells, miR-143 expression was downregulated after shikonin administration, whereas the regulation factor BAG3 was upregulated. Notably, miR-143 overexpression reversed this phenomenon and enhanced the anti-tumor activity of shikonin in glioblastoma stem cells (58). Celastrol is found in the root of *Celastrus orbiculatus*, belonging to the family Celastraceae. It can regulate apoptosis-promoting signaling pathways (75). Celastrol suppresses cellular proliferation and induces apoptosis of ovarian cancer OVCAR3 cells by downregulating miR-21 to inhibit the *PI3K/P-Akt-NF-κB* signaling pathway, thereby revealing a potential therapeutic approach (63).

## Active Ingredients of Chinese Medicine Target miRNAs to Block Tumor Cell Cycle

The occurrence of most tumors is related to the disruption of cell cycle regulation, leading to uncontrolled cell growth. Many active ingredients of Chinese medicine play an anti-tumor role by blocking the tumor cell cycle and inhibiting cell proliferation (**Figure 1**). For instance, in patients with acute lymphoblastic leukemia (ALL), a study demonstrated that the expression of IGFBP3 was decreased in ALL patients. The authors further identified that miR-196b and miR-1290 were overexpressed in T-ALL TALL-104 and B-ALL SUP-B15 cell lines, respectively. As an miR-196b/miR-1290 inhibitor, resveratrol was further demonstrated to exert antitumor effects on ALL cells including cell cycle arrest. Resveratrol blocks T-ALL T-ALL-104 cells during the G1 phase and the B-ALL SUP-B15 cells in the S phase by inhibiting miR-196b/miR-1290 (45). Baicalin upregulates miR-3127-5p, which increases *p21/CDKN1A* and *P27/CDKN1B* expression to inhibit cell proliferation arresting the cell cycle in S and G2/M phases in Bel-7402 cells (43). Celastrol caused G2/M cell cycle arrest that was accompanied by the down-regulation of miR-21 expression. Further study showed that celastrol inhibited p27 protein degradation by inhibiting the miR-21 and mTOR signaling pathways in BGC-823 and MGC-803 cells. The effect of celastrol on cell cycle arrest of gastric cancer cells was due to an increase in the p27 protein level *via* inhibition of the miR-21-mTOR signaling pathway (66).

## Active Ingredients of Chinese Medicine Targeting miRNA Inhibit Tumor Cell Proliferation

**Figure 2:**
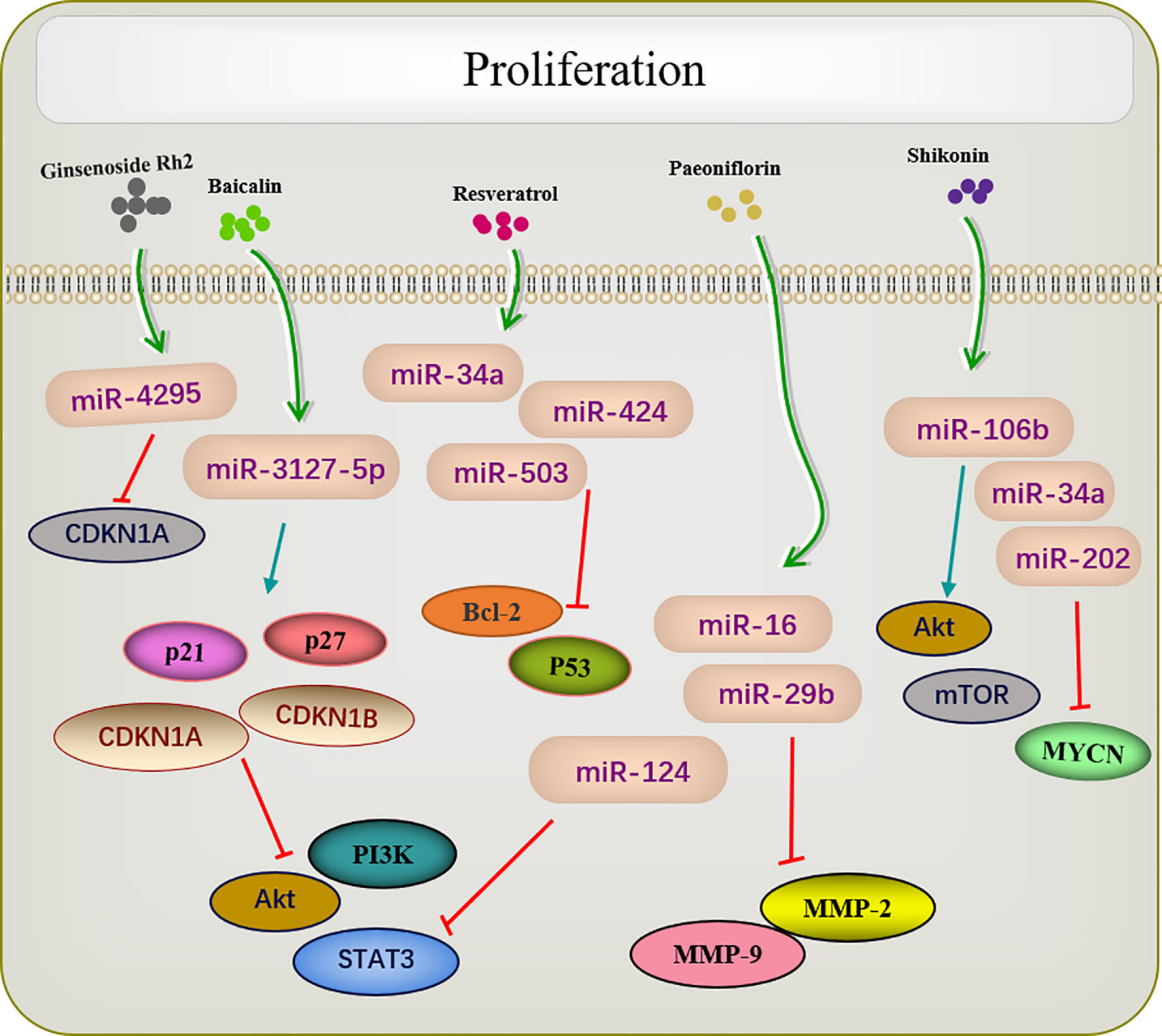
Active ingredients of Chinese medicine targeting miRNAs inhibit tumor cell proliferation.

Many traditional Chinese medicines regulate cell proliferation through miRNAs (**Figure 2**). Paeoniflorin is a monoterpene glycoside with various anti-cancer activities and is derived from *Paeonia lactiflora*. Studies have shown that paeoniflorin has broad-spectrum anti-tumor activities against various cancers (76), including those of liver (77), lungs (78), breast (79), and pancreas (80). miR-124 levels are significantly increased in paeoniflorin-treated MGC-803 cells, which inhibits *PI3K/Akt* and *p-STAT3* expression. A PI3K agonist or *STAT3* overexpression can reverse effects of paeoniflorin on MGC-803 cell proliferation (55).

Shikonin, a natural naphthoquinone isolated from traditional Chinese herbs. miR-106b is one of the most significantly downregulated miRNAs among the several miRNAs dysregulated by shikonin. miR-106b targets the tumor suppressor gene phosphatase and tensin homolog (*PTEN*), thereby modulating *AKT/mTOR* signaling pathway and ultimately inhibiting the proliferation of endometrial cancer cells (59). An earlier report investigated changes in the proliferation of the retinoblastoma cell lines Y-79 and Weri-RB-1 after shikonin administration. The results revealed that shikonin upregulated miR-34a and miR-202 expression and directly targeted the oncogene *MYCN* to degrade its mRNA while inhibiting the proliferation of retinoblastoma cells (62).

Several studies have shown that celastrol can inhibit tumor cell proliferation in several types of cancers. For example, in colon cancer cells, the overexpression of miR-21 enhanced cell viability, inhibited apoptosis, increase Bcl-2 expression, and decreased Bax levels; these effects were reversed by celastrol. As enzymes are involved in cell survival, the PI3K/AKT/GSK-3β pathway provides important signals for tumor cell proliferation Clastrol may inhibit colon cancer cell proliferation by negatively regulating the PI3K/AKT/GSK-3 pathways *via* miR-21 (64). Similar results were reported in lung adenocarcinoma, in which celastrol inhibited cell proliferation and induced apoptosis by regulating the expression levels of miR-24 and miR-181b (69).

Another study showed that ginsenoside Rh2 inhibited the proliferation of prostate cancer cells in a dose-dependent manner, and no *CDKN1A* cell cycle inhibitor was observed in the increased protein of ginsenoside Rh2. Screening all candidate miRNAs for binding to the 3′-untranslated region of *CDKN1A* showed that miR-4295 was dose-dependently inhibited by ginsenoside Rh2. Therefore, comprehensive experimental investigations revealed that ginsenoside Rh2 inhibits prostate cancer cell growth by inhibiting miRNA-4295, which activates *CDKN1A* (37).

## Active Ingredients of Chinese Medicine Targeting miRNA Inhibit Tumor Cell Metastasis and Invasion

**Figure 3:**
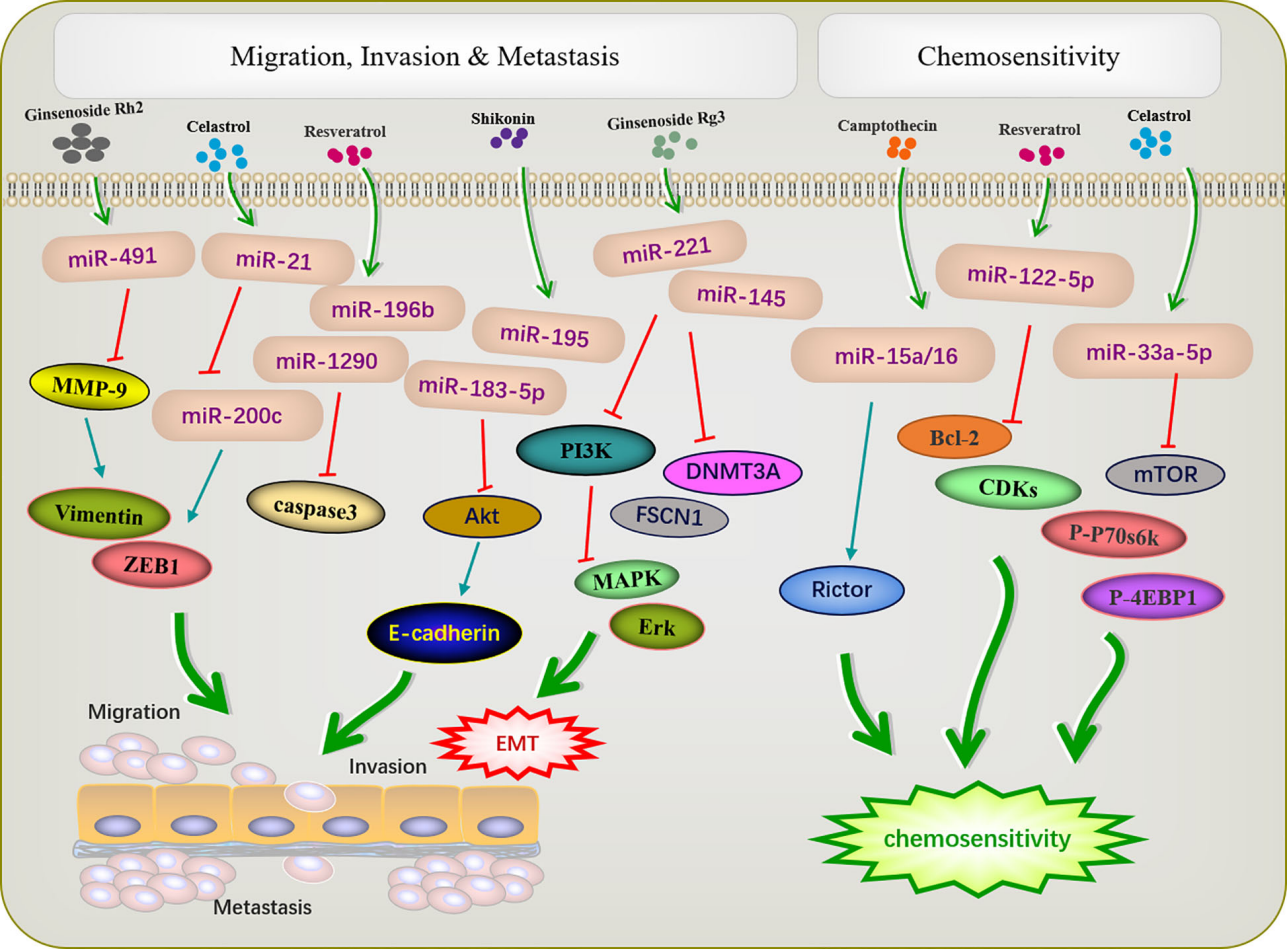
Active ingredients of Chinese medicine targeting miRNAs inhibit tumor cell metastasis and invasion, and reverse tumor cell resistance.

The metastasis and invasion are essential feature characterizing the biological behavior of malignant tumors. The active ingredients of traditional Chinese medicine can inhibit the invasion and metastasis of tumors by regulating miRNA (**Figure 3**). EMT is an important prerequisite for tumor cell metastasis. The miR-200 family inhibits EMT, thereby inhibiting tumor metastasis. A previous study reported that triacetyl resveratrol, a derivative of resveratrol, inhibited pancreatic cancer growth and EMT by upregulating the expression of the members of the miR-200 family and targeting the Shh pathway (48). Moreover, resveratrol increased the expression of miR-200c to inhibit cell proliferation and invasion in HCT116 cells (50). Ginseng saponin Rg3 suppressed EMT in oral squamous cell carcinoma and ovarian cancer *via* miR-221 (51) and miR-145 (52). E-cadherin, the most common EMT protein, plays an important role in tumor invasion, and the loss of E-cadherin expression promotes tumor and EMT. In the cervical cancer cell lines HeLa and C33a, Shikonin inhibits EMT by inducing miR-183-5p expression *via* E-cadherin (61). Similarly, miR-17-5p expression was upregulated in triple-negative breast cancer. The *PTEN* is a direct target of miR-17-5p. Studies have shown that increased expression of *PTEN* can inhibit miR-17-5p and reduce the expression of *Akt* and *P-Akt*, thereby inhibiting EMT and the migration and invasion of triple-negative breast cancer cells (81).

Shikonin can inhibit tumor migration and invasion *via* modulating miRNA-mediated regulation of multiple pathways. miR-195 is an important member of the micro-15/16/195/424/497 family and can be used as a diagnostic biomarker in breast cancer (82). In NCI-N87 cells, shikonin inhibited the proliferation, migration, and invasion by regulating miR-195 to inhibit the *PI3K/AKT* signaling pathway (60). Matrix metalloproteinases (MMPs) play important roles in mediating angiogenesis, metastasis, and invasion. *MMP-2/9* expression is related to the progression of many tumors, such as colon cancer (83), neuroblastoma (84), and bladder cancer (85). Paeoniflorin regulated miR-16/29b, which targeted *MMP-9/2* (56) to inhibit the growth and invasion of multiple myeloma cells (57). Celastrol can downregulate miR-21 and *MMP9* and regulate the expression of the cell migration protein vimentin; this reduces the migration and invasion ability of MKN45 gastric cancer cells (65). Resveratrol significantly inhibited cell migration in T-cell ALL T-ALL-104 and B-cell ALL SUP-B15 cells by inhibiting miR-196b/miR-1290 (45).

## Active Ingredients of Chinese Medicine Targeting miRNA Reverse Tumor Cell Resistance

Increasing evidence has revealed that dysfunctional miRNAs significantly affect chemotherapy resistance. Active ingredients of Chinese medicine play an important role in reducing the toxic and side effects of chemotherapy and improving resistance to chemotherapy (**Figure 3**). A previous study showed that low-invasive breast cancer cells were resistant to amycin and this resistance was reversed by resveratrol, which also targeted the regulatory inhibitor miR-122-5p to influence the cell cycle and apoptosis (49). Increased autophagy during chemotherapy can promote tumor apoptosis or mediate autophagy-related apoptosis. miR-15a and miR-16 effectively induce autophagy, enhancing the therapeutic effect of camptothecin (54). Celastrol reduced *mTOR, P-P70S6K*, and *p-4EBP1* expression by increased miR33a-5p to inhibited tumor growth (70).

## The Active Ingredients of Traditional Chinese Medicine Regulate the Anti-Tumor Mechanism of lncRNAs

Generally, lncRNAs are defined as molecules comprising more than 200 nucleotides lacking protein-coding capacity. However, more recently, lncRNAs have been reported to regulate gene expression (86). Meanwhile, various active ingredients in Chinese medicines, such as curcumin and resveratrol, modulate tumor development *via* lncRNA expression regulation *in vitro* and *in vivo* (**Table 2**). In gemcitabine-resistant pancreatic ductal adenocarcinoma cell, a phenolic compound extracted from turmeric, curcumin, desensitizes chemotherapy-resistant pancreatic ductal adenocarcinoma *via* inhibiting the *PRC2-PVT1-c-Myc* axis. Hence, emerging evidence suggested that curcumin may be an effective sensitizing agent for chemotherapeutic drugs (96). Moreover, curcumin induces the expression of the lncRNA *PINT* to inhibit acute lymphoblastic leukemia cell growth (88).

**Table 2:** Detailed information on Chinese medicine active ingredients targeting lncRNAs.

Active Compound	LncRNA	Cancer	Related Hallmark	Reference
Curcumin	H19	Gastric cancer	Proliferation	(87)
Curcumin	GAS5	Breast cancer	Apoptosis	(12)
Curcumin	PINT	Acute lymphoblastic leukemia	Proliferation	(88)
Curcumin	ROR	Prostate cancer	Proliferation	(31)
Curcumin	PANDAR	Colorectal cancer	Apoptosis	(89)
Curcumin	MEG3	Ovarian cancer	Drug resistance	(90)
Resveratrol	AK001796	Lung cancer	Proliferation/cycle arrest	(91)
Resveratrol	NEAT1	Multiple myeloma	Proliferation/migration	(92)
Matrine	LINC00472	Bladder carcinoma	Growth/metastasis	(93)
Artesunate	UCA1	Prostate cancer	Apoptosis/migration	(94)
Triptolide	THOR	Nasopharyngeal carcinoma	Growth	(95)
Calycosin	EWSAT1	Nasopharyngeal carcinoma	Growth	(30)

Resveratrol is a non-flavonoid polyphenol compound with a wide pharmacological spectrum of anti-cancer, anti-inflammatory, anti-microbial, and antioxidant activity (97). One study on lung cancer reported the upregulation of 21 lncRNAs and downregulation of 19 lncRNAs resveratrol treated A549 cells. Among these, decreased levels of the lncRNA *AK001796* weakened the inhibitory effect of resveratrol on cell proliferation (91). Resveratrol inhibits cell proliferation, migration, and invasion by downregulating *AK001796* and *NEAT1* in lung cancer and multiple myeloma (91, 92). Similarly, triptolide and isoflavone calycosin inhibit tumor cell growth by inhibiting specific lncRNAs in nasopharyngeal cancer. Triptonide inhibits human nasopharyngeal carcinoma cell growth *via* disrupting lncRNA *THOR-IGF2BP1* signaling. Conversely, ectopic lncRNA *THOR* overexpression inhibits Triptonide-induced cytotoxicity in NPC cells (95).

**Figure 4:**
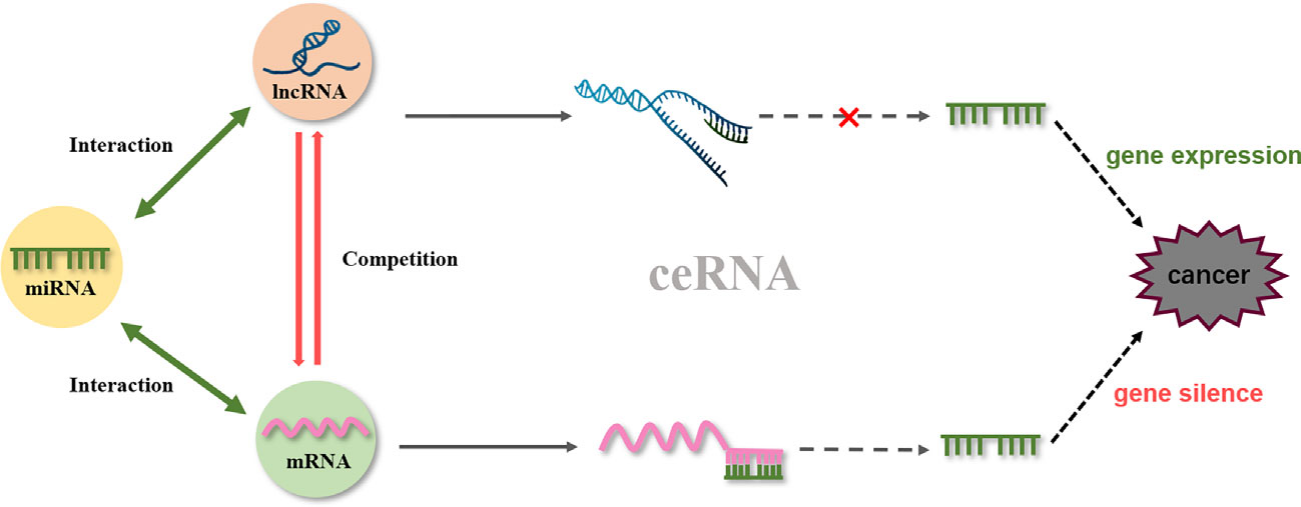
ceRNA network.

One of the representative lncRNAs, *H19*, is recognized as a cancer biomarker and is associated with the occurrence of esophageal cancer (98), colorectal cancer (99), liver cancer (100), breast cancer (101), bladder cancer (102), and stomach cancer. Furthermore, reduced expression of H19 can inhibit cancer development (103, 104). Specifically, curcumin inhibited cell proliferation *via c-Myc/H19* pathway, which reduced the expression of H19 in stomach cancer cells. This indicated that curcumin is a potential drug for gastric cancer (87). Moreover, microarray data identified *H19* as a potential target of Huaier (a fungal parasite on locust trees), the extract from which reduced the expression of *H19*, while also reducing the viability of breast cancer cells by inducing apoptosis *via* regulation of the *H19-miR-675-5p-CBL* axis (105). Besides, certain lncRNAs and miRNAs mutually restrict and regulate target genes to achieve tumor inhibition. For example, in bladder cancer, *H19* can directly bind miR-29b-3p to derepress the target *DNMT3B*. Further, upregulating H19 antagonizes miR-29b-3p-mediated proliferation, migration, and epithelial-mesenchymal transition (EMT) suppression in bladder cells. This evidence demonstrated, for the first time, that *H19* may function as a competing endogenous RNA (ceRNA) for miR-29b-3p and relieve the suppression of *DNMT3B*, leading to EMT and metastasis of bladder cancer (106). In 2011, a new theory was proposed that ceRNAs and miRNA response elements could mediate the interactions between mRNA pseudogenes and some ncRNAs to form a large-scale regulatory network in the transcriptome and serve as a “new language” for “mutual conversation” (107) (**Figure 4**). These networks are characterized by sponge activity, in which ncRNA interacts with the target gene to competitively bind or inhibit. In addition, ceRNAs have been identified as key regulatory factors in cancer (108, 109). *PVT1*, located downstream of the proto-oncogene *Myc* in chromosome 8q24, was used as a ceRNA of miR-216b and miR-152 in non-small-cell lung cancer and osteosarcoma to promote the tumor resistance to anti-cancer drugs (110, 111). In prostate cancer cells, *lncRNA-ROR* and the stem cell marker *Oct4* mRNA contain binding regions for miR-145 and directly compete with this microRNA. Curcumin reduced the expression of endogenous *lncRNA-ROR* and effectively increased the available concentration of miR-145 in human prostate cancer stem cells, where miR-145 prevented cell proliferation by decreasing *Oct4* expression (31). The ceRNA hypothesis has revealed new mechanisms of RNA interactions, which incentivized the analysis of ncRNA role in cancer development.

## Regulation of the Anti-Tumor Mechanism by circRNA and Other ncRNAs

As potential targets for the active ingredients of Chinese medicine, circRNA, siRNA, rRNA, and other non-coding RNAs are involved in tumor development. Some circRNAs affect cancer biogenesis in diverse manners, such as by functioning as miRNA sponges, combining with RNA-binding proteins, acting as transcription factors, and affecting protein translation (112). Matrine decreased circRNA-104075 and Bcl-9 expression significantly *via* inhibition of PI3K/AKT and Wnt-β-catenin pathways,it suppressed cell viability while induceing apoptosis and autophagy in glioma cell line U251 (113). Another study shows that matrine down-regulated the levels of circ_0027345 and HOXD3, and up-regulated miR-345-5p expression. Meanwhile, matrine restrained tumor growth, invasion and promoted autophagy of HCC by regulating the circ_0027345/miR-345-5p/HOXD3 axis *in vivo* (114). Curcumin has antioxidant and anti-cancer properties, and it has also been used as a radiosensitizer. A study compared the differences in circRNA levels in NPC cell lines after radiotherapy and after treatment with curcumin, using a high-throughput microarray. Finally, it was demonstrated by reverse transcription-quantitative polymerase chain reaction assay and wound healing assay that curcumin could enhance radiosensitization of NPC cell lines *via* mediating regulation of tumor stem-like cells by the “hsa_circRNA_102115”-“hsa-miR-335-3p”-“MAPK1” interaction network (115). At present, although circRNA has shown significant activity in the treatment of cancer, there are few reports on the regulation of circRNA by Active Ingredients of Chinese Medicine, which is also an important direction for future researchers to concern and research. circRNA has been expected to become a new molecular biomarker for the clinical diagnosis, treatment and prognosis, and the potential target for targeted therapy. siRNAs are double-stranded RNAs of 20-25 nucleotides and are involved in RNA interference; they regulate gene expression in a specific manner. Multiple synthetic siRNAs can achieve long-term silencing of target genes without interfering with endogenous microRNA pathways. Ginsenoside Rh2 downregulated P-STAT3/STAT3 and intracellular oxidative stress by upregulating PPAR. In response to siRNA-mediated knockdown of PPAR, STAT3 and intracellular oxidative stress were increased (116). rRNA is the most abundant type of RNA in cells. In lung cancer cells, triptolide interrupts rRNA synthesis by inhibiting transcriptional activation of *RNA Pol I* and *UBF*, thereby activating the apoptosis regulators *caspase 9* and *caspase 3* to inhibit *BCL2* and induce apoptosis and cell cycle arrest (117).

## Discussion

During cancer development, abnormal ncRNAs modulate cell proliferation, migration, and invasion by regulating the expression of proto-oncogenes and tumor suppressor genes. Many active ingredients of traditional Chinese medicine, such as resveratrol, matrine, and berberine, have been evaluated *in vivo* and *in vitro* to target specific ncRNA and shown to play anti-cancer roles. This review summarized the finding regarding 16 active ingredients of traditional Chinese medicine that can target miRNAs, lncRNAs, and other ncRNAs, thereby playing an effective role in suppressing cancer growth. Among the ncRNAs regulated by Chinese medicine active ingredients, miR-21 is the most reported ncRNA and is extensively studied in various cancers. It is involved in most of the cancer-related processes, such as cell apoptosis, proliferation, migration, and cell cycle. These findings indicate that miR21 is one of the promising ncRNAs to develop targeted therapeutic agents for many types of cancer. Some active ingredients of traditional Chinese medicine, such as Ginsenoside Rh2 and Resveratrol, promote apoptosis by regulating ncRNAs to target common apoptosis-related target genes, such as *BCL2* and *Caspase3*.

In conclusion, traditional Chinese medicine’s active ingredients significantly ameliorate malignant neoplasms *via* ncRNA regulation, suggesting that active ingredients of traditional Chinese medicine may become alternative therapeutic agents for cancer in the future. At present, most studies have reported that the active ingredients of traditional Chinese medicine mainly target one kind of ncRNA for cancer treatment. However, ceRNA mechanism suggests that several kinds of ncRNA have complex interactions in cancer treatment. Therefore, we need to further explore the detailed anti-cancer mechanism and clinical safety of each of the active ingredients of traditional Chinese medicine. We hope that this review on the regulation of ncRNA by active ingredients of traditional Chinese medicine on tumor will be helpful for future research studies on anti-cancer of traditional Chinese medicine and provide a reference for their clinical application.

## Author Contributions

TL and SH participated in writing, editing, and making figures. ZQ and DL read and approved the final manuscript. All authors contributed to the article and approved the submitted version.

## Funding

This work was supported by the National Natural Science Foundation of China (grant 81973712,81803680,82003985), China Postdoctoral Science Foundation (grant 2020M670825, 2020T130568), Jilin Province Science and Technology Development Project in China (grant 20170309005YY, 20200504005YY), Jilin Province TCM science and technology project (grant 2020041).

## Conflict of Interest

The authors declare that the research was conducted in the absence of any commercial or financial relationships that could be construed as a potential conflict of interest.

## Abbreviations

ncRNA, non-coding RNA; lncRNA, long non-coding RNA; miRNA, microRNA; siRNA, small interfering RNA; piRNA, Piwi-interacting RNA; rRNA, ribosomal RNA; tRNA, transfer RNA; snRNA, small nuclear RNA; EMT, epithelial-mesenchymal transition; ceRNA, competing endogenous RNA; T-ALL, T-cell acute lymphoblastic leukemia; B-ALL, B-cell acute lymphoblastic leukemia; PKM2, pyruvate kinase M2; MMPs, Matrix metalloproteinases; circRNA, circular RNA.
